# Factors Associated with the Occurrence of the First-Follicular-Wave Dominant Follicle on the Ovary Ipsilateral to the Corpus Luteum in Dairy Cattle

**DOI:** 10.3390/ani15152253

**Published:** 2025-07-31

**Authors:** Ryotaro Miura, Motozumi Matsui

**Affiliations:** 1 Department of Large Animal Clinical Sciences, School of Veterinary Medicine, Rakuno Gakuen University, Ebetsu 069-0836, Hokkaido, Japan; r-miura@rakuno.ac.jp; 2 Department of Clinical Veterinary Science, Obihiro University of Agriculture and Veterinary Medicine, Inada, Obihiro 080-8555, Hokkaido, Japan

**Keywords:** dairy cattle, first wave dominant follicle, corpus luteum, location

## Abstract

This study aimed to determine the factors associated with the occurrence of the first-wave dominant follicle (DF) in the ovary ipsilateral to the corpus luteum (CL) in lactating dairy cows and dairy heifers. A total of 505 estruses were investigated (lactating dairy cows, *n* = 361; dairy heifers, *n* = 144). The locations of the preovulatory follicle (PF) and regressed CL were examined at the estrus, and the locations of the first-wave dominant follicle (DF) and newly formed CL were examined seven days after estrus using transrectal ultrasonography. Then, cows were classified into two groups: the first-wave DF in the ovary ipsilateral to the CL (IG) and the first-wave DF in the ovary contralateral to the CL (CG). To evaluate the factors which affect the occurrence of IG and CG, binominal logistic regression analysis was conducted; the location of the PF and regressing CL, season (warm: June–September; cool: October–May), live weight, days in milk at estrus, daily milk production, and body condition score were used as independent variables. The occurrence rate of IG was significantly higher when the PF was located contralateral to the regressing CL (lactating dairy cows, 63.4%; dairy heifers, 58.6%) rather than ipsilateral (lactating dairy cows, 44.9%; dairy heifers, 35.1%). The IG occurrence rate was significantly higher with an increase in daily milk production (<30 kg, 47.3%; 30–40 kg, 55.2%; >40 kg, 60.5%) in lactating dairy cows. In conclusion, the occurrence of IG was associated with relative locations of the PF and regressing CL in lactating dairy cows and dairy heifers and with the level of milk production in lactating dairy cows.

## Simple Summary

The determination of the factors associated with the occurrence of the first-wave dominant follicle (DF) in the ovary ipsilateral to the corpus luteum (CL) (IG) in lactating Holstein dairy cows and dairy heifers was conducted in this study. The IG occurrence rate was significantly higher when the preovulatory follicle (PF) was located contralateral to the regressing CL rather than ipsilateral in lactating dairy cows and heifers. In addition, the IG occurrence rate increased with the increasing of milk production. From these results, the first-wave DF and CL locational development was associated with the PF and CL locational relationship and with the level of milk production in dairy cattle.

## 1. Introduction

In cattle, two or three follicular waves occur during the estrous cycle [1,2,3,4]. The first follicular wave emerges soon after estrus, and the first-wave dominant follicle (DF) undergoes atresia during 8 to 10 days of the estrous cycle [1,2,3,4]. The dynamics of DF development and the hormonal milieu during first-wave development have been well studied [5,6,7]. In a previous study, we found that the development of the first-wave DF in the ovary ipsilateral to the CL was associated with reduced conception rates in lactating dairy cows [8,9] and dairy heifers [8]. Thus, the relative location of follicle and CL must be an important factor affecting fertility in cattle. In addition, the effect of hCG treatment five days after artificial insemination on conception rate were different between the first-wave DF in the ovary ipsilateral and contralateral to the CL/hCG administration improve the conception rate only to cows with the first wave DF ipsilateral to the CL [9]. However, the locational relationship of the first-wave DF on fertility and factors which were associated with the development of the first-wave DF in the ovary ipsilateral or contralateral to the corpus luteum (CL) has not been fully investigated in dairy cattle. Although there are multiple factors which affect the herd level fertility, such as types of estrus/ovulation synchronization program [10], herd management [11], heat stress [12], diseases [13,14], prepartum body condition score (BCS) changes [15,16] it is necessary to evaluate the factors related to ovarian physiology that affect reproductive performance. A better understanding of the reproductive function of the cow at the farm level would improve the health and welfare of the dairy cow, favoring both increased productivity and better use of land resources.

A previous study on heifer research demonstrated that the PF located contralateral to the regressing CL resulted in ipsilateral first-wave DF and CL (94%), while the PF ipsilateral to the regressing CL resulted in contralateral first-wave DF and CL (75%) in dairy heifers [17]. This observation suggests that the development of the first-wave DF is influenced by the relative location of the PF and the regressing CL.

In lactating dairy cows, the ovary ipsilateral to the side of previous pregnancy a lower functional activity than that exhibited by the contralateral ovary until the second postpartum ovulation [18,19]. However, these locational relationships have not been investigated in lactating dairy cows during the breeding period. Although negative energy balance and milk production level are factors known to affect fertility [15,20], no studies have described the relationship between the occurrence of the first-wave DF in the ovary ipsilateral to the CL and specific physiological factors such as BCS, milk production and parity. Therefore, to determine the cause of lower fertility in the first-wave DF located ipsilateral to the CL, we need to identify factors associated with the development of the first-wave DF on the ovary ipsilateral to the CL. By clarifying the factors related to the locational relationship of the first-wave DF and CL in the ovaries; to evaluate the relationship between the DF and CL in a herd level, it may be possible to propose improvement measures for the entire herd or individual cows from a nutritional perspective. Furthermore, it is expected that this information about the ovarian condition of cows in a herd may provide important insights for developing hormonal treatment strategies after artificial insemination.

The objective of our study was to identify the factors associated with the development of the first-wave DF in the ovary ipsilateral to the CL in lactating dairy cows and dairy heifers.

## 2. Materials and Methods

### 2.1. Animals and Management

Postpartum lactating Holstein cows (*n* = 236) and heifers (*n* = 92) at the Field Science Center of Obihiro University in Hokkaido, northeast Japan, were used; a single evaluation was conducted for each cow. The study was conducted from March 2008 to February 2015. Cattle underwent regular estrous cycles and were clinically healthy during the breeding period. Cattle experiencing reproductive or metabolic diseases were excluded. In total, 505 estruses were recorded, including 361 estruses in lactating dairy cows (postpartum day of estrus, 113.9 ± 2.9; parity, 2.0 ± 0.1; milk production, 34.4 ± 0.4 kg/d; BCS, 2.88 ± 0.02; live weight, 637.4 ± 3.9 kg; mean ± SEM) and 144 estruses in dairy heifers (age, 14.1 ± 0.2 months; live weight, 433.3 ± 4.4 kg). The cows were kept in a free-stall barn under the normal management program of the Field Science Center of Obihiro University and were fed a TMR diet consisting of corn silage, grass silage, soybean meal, corn grain, and concentrate, with free access to water. All cows were milked twice daily on a parallel parlor.

### 2.2. Estrus Detection and Ovary Examination

Estrus was detected by visual observation, examination of tail paint, and transrectal ultrasonography to confirm the PF and regressed CL. Ovulation was confirmed using transrectal ultrasonography which equipped with a 5.0 MHz linear transducer (HS-101V, Honda Electronics, Toyohashi, Japan) every 24 h after estrus detection. In addition, the location of the PF in the ovary was confirmed at that time to be ipsilateral (Pre-IG) or contralateral (Pre-CG) to the regressed CL. After ovulation was confirmed, we checked the CL and the first-wave DF (diameter ≥ 1.2 cm) 5 or 6 d after estrus using transrectal ultrasonography. If the largest follicle was <1.2 cm, the ovary was re-examined by transrectal ultrasonography 2 to 3 d later, and the largest follicle was defined as the first-wave DF. If growth of the largest follicle was not evident, the animal was excluded from the study. In addition, if an animal had co-dominant follicles (more than two follicles with diameter ≥1.2 cm), it was excluded from the study. The location of the first-wave DF in the ovary was confirmed at that time to be ipsilateral (IG) or contralateral (CG) to the CL.

### 2.3. Statistical Analysis

Binary logistic regression analysis was applied to data from lactating dairy cows and heifers, respectively. The occurrence of IG and CG was analyzed as a dependent variable (IG: 1, CG: 0). The common independent variables were the location of the PF and regressed CL (Pre-IG, Pre-CG), and season (warm: June–September, cool: October–May). In heifers, the additional independent variable of live weight (<441 kg, ≥441 kg) was used in the logistic regression model. In lactating cows, postpartum day of estrus (≤120 d, >120 d), milk production (<30 kg, 30–40 kg, >40 kg), parity (1, 2, ≥3), BCS (<3.00, ≥3.00) [21], and live weight (<640 kg, ≥640 kg) were incorporated into the logistic regression model. Live weight, milk production, and BCS were measured monthly during experimental period. In the analysis, we used the values obtained in the month in which the animal was in estrus. Cochran-Armitage test was used to investigate the relationship between the occurrence of IG and milk production level (<30 kg, 30–40 kg, >40 kg) in lactating dairy cows. Baseline variables with *p* < 0.20 in univariate analysis were included in the multivariate models. Statistical significance was defined as *p* < 0.05. All statistical analyses were performed using EZR (Saitama Medical Center, Jichi Medical University, Saitama, Japan), graphics user interface for R (The R foundation for Statistical Computing, Vienna, Austria). This software is a modified version of R Commander designed to add statistical functions frequently used in biostatistics [22].

## 3. Results

### 3.1. Factors Affecting the Occurrence of IG in Lactating Dairy Cows

Logistic regression analysis revealed that the locations of PF and regressed CL (*p* < 0.001) and milk production (*p* < 0.05) were significantly associated with the occurrence of IG (Table 1). The occurrence rate of IG was greater when the PF and the regressing CL were located contralateral [63.4% (111/174)], and lower when the PF and regressing CL were located ipsilateral [44.9% (84/187)] (Table 2). The IG occurrence rate increased with higher milk production [<30 kg, 47.3% (52/110); 30–40 kg, 55.2% (91/165); >40 kg, 60.5% (52/86); *p* < 0.05] (Table 3).

Season, postpartum day of estrus, BCS, and live weight were not associated with the occurrence of IG in dairy heifers (Table 4).

### 3.2. Factors Affecting the Occurrence of IG in Dairy Heifers

Logistic regression analysis revealed that the location of the PF and regressed CL was significantly associated with the occurrence of IG (*p* < 0.01) (Table 1). The IG occurrence rate was greater when the PF and the regressing CL were located contralateral [58.6% (51/87)] and lower when the PF and regressing CL were located ipsilateral [35.1% (20/57)] (Table 2).

Season and live weight were not associated with the occurrence of IG in dairy heifers (Table 4).

## 4. Discussion

The present study found that the relative locations of the PF and the regressed CL were associated with the occurrence of IG and CG in lactating dairy cows and dairy heifers. Milk production was also positively associated with the IG occurrence rate in lactating dairy cows. To the best of our knowledge, this study is the first to investigate factors that affect the occurrence rate of first-wave DF in the ovary ipsilateral to the CL in lactating dairy cows and dairy heifers.

In a previous study, the PF contralateral to the regressing CL resulted in ipsilateral first-wave DF and CL, while the PF ipsilateral to the regressing CL resulted in contralateral first-wave DF and CL in heifers [17]. The present study confirms these results, not only in daily heifers but also in lactating dairy cows. During luteolysis, blood flow perfusion to the ovary contains a regressing CL decrease and the number of small antral follicles (follicles of 2.0 mm) lower with ovary bearing regressing CL than ovary without regressing CL in dairy heifers [23]. In addition, even after the ovulation, the numbers of antral follicles (follicles of 6.0 mm) were lower in ovary which had regressed CL than opposite side of ovary [23]. From these results, Antral follicle development may be suppressed by the ovary containing a regressing CL, resulting in more frequent DF developed in the contralateral ovary.

We observed that the IG occurrence rate increased with higher milk production in lactating dairy cows. Milk production level affects the incidence of multiple ovulations [24,25,26], plasma estradiol concentration at estrus, plasma P_4_ concentration, and steroid hormone metabolism [25,26]. Thus, one factor affecting the occurrence of IG or CG in lactating dairy cows may be their metabolic status. These results suggested that ovarian characteristics at estrus, milk production level affect the occurrence of IG or CG. From the previous study, the factors which affect the bias of the side of the ovary on which the DF develop have been reported in postpartum lactating dairy cows; such as the effect of the side opposite that of the preceding pregnancy [18,19], and the degree of bacterial infection in the uterus [27]. However, there are no reports evaluating factors affecting the locational relationship of the first-wave DF and CL in mid-lactation period. Kawashima reported that when the blood glucose concentration and BCS were compared between the cows with ovulation or anovulation of the DF of the first follicular wave post-partum, blood glucose concentration and BCS were higher in cows with ovulation of the DF of the first follicular wave post-partum [28]; from this results, There is suggested that DF development and function are influenced by blood glucose concentration and BCS status. Kida reported that there are negative correlations between milk yield and BCS or blood glucose concentration in mid-lactation period in lactating Holstein dairy cows [29]. Therefore, it was speculated that the low blood glucose concentration and BCS in the high milk producing cows in this study, in other words the low energy status, may have biased DF development to one ovary.

From this study, the occurrence rates of IG were not associated with the season, postpartum days of estrus, BCS, and live weight in lactating dairy cows and heifers. From these results indicated that it was speculated that the degree of growth, body size, and fat accumulation during the estrous cycle do not affect the occurrence of IG, and that the metabolic load caused by the milk yield of each cow has a greater effect than the postpartum days from calving on the occurrence rate of IG. Based on these results, since the metabolic state of cows also affects the occurrence of IG which cows were thought with low reproductive performance, it is expected that evaluating the locational relationship between the CL and DF in the ovaries as a herd will enable improvements in nutritional management and the consideration of selection of hCG treatment after AI in cases where IG increases.

The physiological mechanisms underlying these results were not fully clarified in this study. Further research is required to determine the mechanism whereby IG occurs, particularly with respect to the number of follicular waves, the effect of the ovary side (left and right), serum biochemical parameters (such as glucose, non-esterified fatty acid, β-hydroxybutyrate) and milk production in mid-lactation period. In addition, this study has several limitations. Our data was collected from animals living at only one farm, so we could not exclude bias related to the characteristics of the farm. Therefore, these relationships must be analyzed using large numbers of herds.

## 5. Conclusions

In conclusion, the present study demonstrates that the factor associated with the occurrence of the first-wave DF on the ovary ipsilateral to the CL in these cattle was the relative locations of the PF and regressing CL. The level of milk production was also associated with the IG occurrence rate in lactating dairy cows.

## Figures and Tables

**Table 1:** Regression table of the final logistic regression model for factors affecting occurrence of IG ^1^ in lactating dairy cows and dairy heifers.

Group	Coefficient	Odds Ratio	95% CI	*p*-Value
Lactating dairy cows				
Intercept	−0.817			
Location ^2^	0.817	2.260	1.490–3.450	<0.001
Milk production ^3^	0.309	1.360	1.020–1.820	<0.05
Dairy heifers				
Intercept	−0.615			
Location	0.964	2.620	1.310–5.230	<0.01

^1^ IG = Ipsilateral group. The first-wave dominant follicle located ipsilateral to the CL. ^2^ Location = the location of the preovulatory follicle in the ovary was confirmed to be ipsilateral or contralateral to the corpus luteum at estrus. ^3^ Milk production = Daily milk yield which was measured monthly during the experiment period. Values which was measured the month when cows expressed estrus.

**Table 2:** The locational effect of preovulatory follicle and CL on occurrence rates of IG ^1^ in lactating dairy cows and heifers.

IG Occurrence Rate (%)	Pre-IG ^2^	Pre-CG ^3^	*p*-Value
Lactating dairy cows	44.9	63.4	<0.001
	(84/187)	(111/174)	
Dairy heifers	35.1	58.6	<0.01

^1^ IG = Ipsilateral group. The first-wave dominant follicle located ipsilateral to the CL.^2^ Pre-IG = the location of the PF in the ovary was confirmed at that time to be ipsilateral to the regressed CL. ^3^ Pre-CG = the location of the PF in the ovary was confirmed at that time to be contralateral to the regressed CL.

**Table 3:** Occurrence rates among different milk production level in lactating dairy cows.

IG ^1^ Occurrence Rate (%)	<30 kg	30–40 kg	>40 kg	*p*-Value ^2^
Lactating dairy cows	47.3	55.2	60.5	<0.05

^1^ IG = Ipsilateral group. The first-wave dominant follicle located ipsilateral to the CL; ^2^ The results of Cochran-Armitage test.

**Table 4:** The factors which were not associated with the occurrence rates of IG ^1^ in lactating dairy cows and heifers.

Factors	Group	*n*	IG Occurrence Rate (%)	*p*-Value
Lactating dairy cows				
Season	Warm	109	50.5	0.340
	Cool	254	56.0	
Postpartum days of estrus	≤120 d	228	54.8	0.783
	>120 d	135	53.3	
BCS ^2^	<3.00	166	56.0	0.538
	≥3.00	197	52.8	
Live weight	<640 kg	185	52.4	0.438
	≥640 kg	177	56.5	
Dairy heifers				
Season	Warm	37	40.5	0.291
	Cool	107	52.3	
Live weight	<441 kg	77	53.2	0.311
	≥441 kg	67	44.8	

^1^ IG = Ipsilateral group. The first-wave dominant follicle located ipsilateral to the CL. ^2^ BCS = Body condition score.

## Data Availability

The data presented in this study are available on request from the corresponding author.
